# The G-patch protein Cwf28 interacts with the RNA helicase Cdc28 in catalytically active spliceosomes in *Schizosaccharomyces pombe*

**DOI:** 10.1371/journal.pone.0357276

**Published:** 2026-09-03

**Authors:** Laura Olivia Karika, Ingrid Cipakova, Miroslava Kretova, Tomas Selicky, Natalia Cmikova, Daniela Nemcekova, Lenka Kohutova, Peter Barath, Lubos Cipak

**Affiliations:** 1 Cancer Research Institute, Biomedical Research Center, Slovak Academy of Sciences, Bratislava, Slovakia; 2 Department of Medical Biochemistry and Biophysics, Umeå University, Umeå, Sweden; 3 Institute of Chemistry, Slovak Academy of Sciences, Bratislava, Slovakia; Centre de Recherche en Biologie cellulaire de Montpellier, FRANCE

## Abstract

G-patch proteins are emerging as key regulatory cofactors of DEAH-box RNA helicases involved in pre-mRNA splicing, yet the functions of many family members remain poorly understood. Here, we characterize the conserved G-patch domain–containing protein Cwf28 from *Schizosaccharomyces pomb*e, an essential factor with a previously unclear molecular function, and define its role within the spliceosome. Tandem affinity purification coupled with mass spectrometry revealed that Cwf28 predominantly associates with components of the Prp19 complex (NTC) and factors involved in the catalytic activation and progression of the spliceosome, placing it within catalytically active spliceosomal assemblies. Gene ontology analysis showed enrichment of the Cwf28 interactome in factors involved in spliceosome assembly, activation, and catalytic remodeling. Notably, the DEAH-box RNA helicase Cdc28, the ortholog of human DHX16 and *Saccharomyces cerevisiae* Prp2, was identified as the most abundant interactor. Further analysis demonstrated that Cwf28 interacts with Cdc28 via its conserved G-patch domain, and domain mapping confirmed that this interaction is G-patch domain dependent. Together, these findings identify Cwf28 as a component of catalytically active spliceosomes and suggest a potential role for Cwf28 in modulating the activity of the RNA helicase Cdc28, providing insight into conserved mechanisms underlying RNA helicase regulation during splicing.

## Introduction

Pre-mRNA splicing removes introns from precursor transcripts and ligates exons to form mature messenger RNA (mRNA). This process is catalyzed by the spliceosome, a highly dynamic ribonucleoprotein complex composed of small nuclear ribonucleoproteins (snRNPs) and numerous auxiliary proteins [1]. Splicing is initiated by recognition of the 5′ splice site and branch point by early spliceosomal components, forming an initial assembly complex. ATP-dependent recruitment of additional snRNPs then occurs, promoting progression from the early (E) commitment complex to the assembly of the precatalytic B complex. Extensive rearrangements then drive spliceosome activation, including remodeling of RNA–RNA and RNA–protein interactions that position the splice sites for catalysis. The first transesterification reaction cleaves the 5′ exon and forms a lariat intermediate, while the second reaction ligates the exons and releases the intron. Finally, the spliceosome is disassembled, allowing its components to be recycled for subsequent rounds of splicing [1,2].

In the fission yeast *Schizosaccharomyces pombe*, a substantial fraction of genes contains multiple introns with splice-site features resembling those of metazoans. Spliceosome assembly in this organism follows an evolutionarily conserved pathway, progressing from E complex formation through recruitment of the U2 snRNP and the U4/U6 × U5 tri-snRNP to yield a catalytically competent spliceosome. Genome-wide analyses in *S. pombe* suggest relatively rapid and less stringent early spliceosome assembly, with fidelity control mechanisms acting during initial splice-site recognition, as observed in other eukaryotes [3–6]. For example, the Gpl1–Gih35 complex (homologous to the human GPATCH1–DHX35 complex) has been shown to associate with aberrant spliceosomes in *S. pombe*, where it inhibits splicing progression and promotes their targeting to a discard pathway, thereby contributing to spliceosome quality control [7–9].

RNA helicases play central roles in regulating pre-mRNA splicing by driving the dynamic rearrangements required for spliceosome assembly, activation, and fidelity control [10]. In human cells, helicases such as DHX16, SNRNP200, and DHX38 act in a coordinated manner to promote structural transitions within the spliceosome, including the release of proofreading factors, repositioning of spliceosomal components, and formation of the catalytically active RNA network [2,11,12]. Similarly, in *Saccharomyces cerevisiae*, the helicase Prp2 (orthologous to DHX16) remodels the B^*act*^ complex to generate the catalytically competent B* complex required for the first catalytic step [13,14]. In *S. pombe*, the helicase Cdc28 is predicted to be the ortholog of DHX16/Prp2 [15], although its precise role in the catalytic cycle remains to be fully defined.

G-patch proteins have emerged as critical regulatory cofactors that modulate the activity of RNA helicases involved in pre-mRNA splicing and ribosome biogenesis [10,14,16–19]. In human cells, several DEAH-box RNA helicases interact with G-patch domain–containing proteins, often in a selective manner. For example, DHX16 and DHX35 appear to associate predominantly with single G-patch partners, GPKOW [20] and GPATCH1 [9], respectively, whereas DHX15 exhibits a broader interaction spectrum, forming complexes with multiple G-patch proteins, including GPATCH2, GPATCH3, GPATCH8, RBM5, SUGP1, TFIP11, and ZGPAT [17,18,21–26]. These interaction patterns underscore both the specificity and versatility of RNA helicase regulation and reflect their participation in multiple RNA processing pathways and distinct stages of spliceosome assembly and remodeling.

Consistent with these observations, regulatory interactions between spliceosomal RNA helicases and G-patch proteins are conserved in yeast. In *S. cerevisiae*, the G-patch proteins Ntr1 and Cmg1 associate with Prp43, the functional ortholog of human DHX15, and contribute to its regulation [27,28]. In contrast, the G-patch protein Spp2 is an important cofactor of the DEAH-box helicase Prp2, binding its C-terminal region through the G-patch domain and stimulating Prp2 ATPase activity. Although Spp2 is not required for Prp2 recruitment or ATP hydrolysis within the B^*act*^ spliceosome, it is essential for coupling Prp2 activity to productive spliceosomal remodeling and the transition from the B^*act*^ to the catalytically active B* state [13,14,29,30]. In *S. pombe*, analogous relationships have been described, including the functional coupling of Ntr1 and Gpl1 with the DEAH-box helicases Prp43 and Gih35, respectively, contributing to spliceosome disassembly, remodeling, and proofreading [7,8,31]. More recently, the *S. pombe* G-patch protein Sap34 (ortholog of human GPATCH11) has been identified in early spliceosomal complexes, where it associates with Prp43 and may regulate its proofreading activity [32]. Together, these findings highlight the evolutionarily conserved role of G-patch proteins as key modulators of spliceosomal RNA helicases.

In humans, the orthologous protein GPKOW has been proposed to function as a cofactor of DHX16 [10,17,18,20], but the mechanistic evidence supporting this interaction remains less extensive than that established for the *S. cerevisiae* Spp2–Prp2 pair. In *S. pombe*, the poorly characterized G-patch protein Cwf28 (ortholog of GPKOW/Spp2) has been reported to co-purify with components of the Prp19 complex (NTC), including Cdc5, as well as the G10 protein Cwf14 [33,34], but its molecular function and interaction partners remain largely unknown.

To elucidate the molecular function of the conserved and essential G-patch domain–containing protein Cwf28 in *S. pombe*, we performed affinity purification of Cwf28-containing complexes followed by comprehensive interactome analysis. This analysis revealed the RNA helicase Cdc28 as a major interactor of Cwf28, along with components of the NTC and factors involved in the catalytic activation and progression of the spliceosome. Subsequent protein–protein and domain interaction analyses demonstrated that Cwf28 associates with Cdc28 via its conserved G-patch domain, a motif commonly implicated in modulating RNA helicase activity. Collectively, these findings support a model in which Cwf28 associates with Cdc28 during pre-mRNA splicing and suggest a potential role for this interaction in regulating Cdc28 function.

## Materials and methods

### Strains and media

The *S. pombe* strain expressing TAP-tagged Cwf28 protein (*h*^*-*^ *cwf28-TAP::KanMX4*) was generated as described previously [35]. The primers used to construct the tagging plasmid are listed in S1 Table. Cells were grown in complete YE + 5S medium at 32 °C (5 g/L yeast extract, 3% (w/v) glucose, 0.1 g/L L-leucine, 0.1 g/L L-lysine, 0.1 g/L L-histidine, 0.1 g/L uracil, and 0.15 g/L adenine). For a solid medium, 2% (w/v) agar was added. During selection, geneticin was added to the YE + 5S medium at 150 µg/mL. The *E. coli* strain BTH101 was cultured at 30 °C in Luria–Bertani (LB) medium, composed of 10 g/L tryptone, 5 g/L yeast extract, and 10 g/L NaCl.

### Tandem affinity purification

Cells expressing Cwf28-TAP protein were grown in 14 L of YE + 5S medium at 32 °C to mid-log phase (OD_595_ = 0.7–0.8) and harvested by centrifugation (4,000 × g, 10 min, 4 °C). Cells were frozen in liquid nitrogen, and yeast cell powder (30 g) was generated using a SPEX SamplePrep 6770 Freezer/Mill (SPEX SamplePrep). Proteins were extracted in IPP150 buffer (50 mM Tris-HCl, pH 8.0; 150 mM NaCl; 10% glycerol; 0.1% NP-40). The buffer was supplemented with 1 mM PMSF, protease inhibitors (cOmplete, EDTA-free; Roche), and phosphatase inhibitor mix (20 mM NaF; 5 mM Na-pyrophosphate; 10 mM β-glycerophosphate; 1 mM Na_3_VO_4_). Yeast cell powder was resuspended at a ratio of 1 g per 4 mL of IPP150 buffer. The protein extract was clarified by centrifugation (40,000 × g, 3 × 10 min, 4 °C) and subjected to tandem affinity purification as described previously [36]. Briefly, 400 µL of IgG Sepharose 6 Fast Flow beads were washed in IPP150 buffer and incubated with protein extract for 2 h at 4 °C with gentle rotation. Subsequently, the beads with bound proteins were washed with 20 bead volumes of IPP150 buffer and equilibrated with five bead volumes of TEV cleavage buffer (TCB: 10 mM Tris-HCl, pH 8; 150 mM NaCl; 10% glycerol; 0.1% NP-40; 0.5 mM EDTA; 1 mM DTT). On-bead cleavage was performed in 2 mL of TCB supplemented with 400 U of Turbo TEV protease for 2 h at 16 °C. The resulting eluate was supplemented with 6 µL of 1 M CaCl_2_ and diluted with 6 mL of Calmodulin Binding Buffer 1 (CBB1: 10 mM Tris-HCl, pH 8; 150 mM NaCl; 10% glycerol; 0.1% NP-40; 1 mM imidazole; 1 mM Mg(OAc)_2_; 2 mM CaCl_2_; 10 mM β-mercaptoethanol). Subsequently, 100 µL of Calmodulin Sepharose 4B beads washed in CBB1 were added, and binding was allowed for 2 h at 4 °C with gentle rotation. Beads were washed with 10 bead volumes of CBB1 followed by five bead volumes of Calmodulin Binding Buffer 2 (10 mM Tris-HCl, pH 8; 150 mM NaCl; 1 mM Mg(OAc)_2_; 2 mM CaCl_2_; 1 mM β-mercaptoethanol). Proteins were eluted stepwise using one bead volume of elution buffer (10 mM Tris-HCl, pH 8; 150 mM NaCl; 1 mM Mg(OAc)_2_; 2 mM EGTA; 1 mM β-mercaptoethanol). Protein eluates were analyzed by 8% SDS–PAGE and visualized by silver staining [37]. Protein-containing eluates were pooled and subjected to LC–MS/MS analysis.

### Mass spectrometry analysis

The samples were reduced with 5 mM DTT, then alkylated with 15 mM iodoacetamide, and the reaction was quenched with an additional 5 mM DTT. Digestion with trypsin (1:25 w/w) was performed overnight (16 h) in the presence of phosphatase inhibitors at 37 °C. The microtip C18 SPE was used to purify the peptides. For liquid chromatography-coupled mass spectrometry on a Dionex UltiMate 3000 RSLC nano system (Thermo Scientific) and Orbitrap Elite mass spectrometer (Thermo Scientific) peptides were loaded onto a PepMap100 C18 trap column (300 μm × 5 mm, 5-μm particle size, Thermo Scientific), separated with the EASY-Spray PepMap RLSC C18 analytical column with an integrated nanospray emitter (75 μm × 500 mm, 2-μm particle size, Thermo Scientific) using a 60-min gradient (2.4%–34.4% acetonitrile) at a flow rate 250 nL/min and sprayed directly into the mass spectrometer. Precursors were measured in the mass range 300–1,700 m/z with a resolution of 120,000 and selected for fragmentation in a data-dependent mode using the Top15 strategy. The fragmentation was performed by the HCD mechanism with a normalized collision energy of 25%, and MS/MS scans were acquired with a resolution of 15,000. Each sample was measured in technical duplicates. The datasets were processed by MaxQuant [38] version 2.7.0.0. Carbamidomethylation (C) was set as a permanent modification, and oxidation (M), acetylation (protein N-terminus), and phosphorylation (STY) were set as variable modifications. The search was performed against *S. pombe* proteomes from UniProt (downloaded 9.3.2026, isoforms included) and PomBase (downloaded 11.3.2026). Identified proteins and phosphosites with localization probability > 0.75 are listed in the S1 Table. Proteins are arranged in descending order from the highest iBAQ value (intensity normalized in order to be proportional to copy number and not to protein mass). To assess whether the identified phosphosites may represent potential CK2 target sites, phosphorylation site predictions were performed using NetPhos 3.1 [39].

### Functional analysis of Cwf28 interactome

Gene Ontology (GO) term enrichment was performed using the STRING database [40]. The protein–protein interaction network among proteins co-purified with Cwf28 was constructed with STRING v12.0 (http://string-db.org) using the high-confidence setting (0.7).

### Mutagenesis of the *cwf28* allele

PCR-based random mutagenesis was used to introduce mutations within the *cwf28* allele [41]. Mutagenesis was performed using 50 ng of *cwf28*-pUT18 plasmid as the template in a 50 μL reaction containing 1 × Standard Taq buffer (10 mM Tris-HCl, pH 8.3, 50 mM KCl, 1.5 mM MgCl_2_), supplemented with an additional 7 mM MgCl_2_, 1 mM each of dCTP and dTTP, 0.2 mM each of dATP and dGTP, 2 μM of primers (5′-CGACAGGTTTCCCGACTGG-3′ and 5′-GAAGTTACAGAAGTACTCGTACACTTCCCCGGG-3′), 0.05 U Taq DNA polymerase, and 0.5 mM MnCl_2_ added immediately before PCR. Thermal cycling conditions were as follows: initial denaturation at 95 °C for 3 min, 12 cycles of 95 °C for 1 min, 56 °C for 1 min, and 68 °C for 3 min, followed by a final extension at 68 °C for 10 min. Following mutagenesis, PCR products were digested with SmaI and PstI and cloned into the pUT18 vector. Plasmids from positive transformants were isolated and sequenced to identify mutations in the *cwf28* allele. Subsequently, plasmids encoding single amino acid substitutions within the G-patch domain were used for protein–protein interaction analysis.

### Protein–protein interaction analysis

The sequences encoding the full-length ORFs of Cdc28, Cwf28, specific Cwf28 constructs, or G-patch domains engineered to encode defined single amino acid substitutions were created using specific primers (S1 Table) and subcloned or assembled in-frame with the T18 and T25 fragments of adenylate cyclase into the pUT18 and pKNT25 BACTH vectors using the NEBuilder HiFi DNA Assembly Cloning Kit [42]. The prepared plasmids were propagated in *E. coli DH5α*, purified, and co-transformed into *E. coli BTH101* (10 ng of each plasmid). Positive transformants were screened on LB plates supplemented with 40 µg/mL X-gal, 0.5 mM IPTG, 100 µg/mL ampicillin, and 50 µg/mL kanamycin (at 30 °C). Transformants were then spotted onto fresh LB plates supplemented with 40 µg/mL X-gal, 0.5 mM IPTG, 100 µg/mL ampicillin, and 50 µg/mL kanamycin, and incubated for 24 h at 30 °C.

### β-galactosidase activity assay

β-galactosidase activity assay was measured at 30 °C on stationary-phase aliquots of cultures in 3 mL of LB medium in the presence of 0.5 mM IPTG and antibiotics as described previously [43]. The optical density (OD_600_) of each culture was recorded. Cells were permeabilized by adding 15 µL of toluene and 15 µL of 0.1% SDS solution to 1.25 mL of culture, vortexed, plugged with cotton, and vigorously agitated for 40 min at 37 °C. Aliquots (0.1 mL) of permeabilized cells were mixed with 0.9 mL of PM2 assay buffer (60 mM Na_2_HPO_4_, 40 mM NaH_2_PO_4_, 0.14 mM MnCl_2_, and 1 mM MgSO_4_, pH 7.0) supplemented with 7 µL of β-mercaptoethanol (final concentration 100 mM) and preincubated for 5 min at 30 °C. PM2 assay buffer served as a blank. The reaction was initiated by adding 200 µL of 0.4% (w/v) ONPG, followed by incubation at 30 °C. The reaction was allowed to proceed until a visible yellow color developed, and the reaction time was recorded. The reaction was then terminated by adding 200 µL of 1 M Na_2_CO_3_. OD_420_ and OD_550_ were measured with an S-200 Spectrophotometer (Boeco, Germany). β-galactosidase activity was expressed as Miller units using the following equation: Miller Units = 1000 × [(OD_420_–1.75 × OD_550_)]/(*t* × V × OD_600_), where OD_420_ is the absorbance of the yellow *o*-nitrophenol, OD_550_ is the scatter from cell debris, which, when multiplied by 1.75 approximates the scatter observed at 420 nm, OD_600_ reflects cell density, *t* is reaction time in min and V is a volume of culture assayed in mL. The Miller Units give the change in OD_420_/min/mL of cells/OD_600_.

### Statistical analysis

Statistical significance of interactions between Cdc28, Cwf28, and Cwf28 G-patch domain constructs was evaluated relative to the full-length Cwf28 construct using an unpaired *t*-test in Microsoft Excel. *p*-values <0.05 were considered statistically significant.

### AlphaFold modeling

The amino acid sequences of Cdc28 and Cwf28 were used as input for AlphaFold-Multimer to predict the structure of their protein complex [44,45]. The predicted model was subsequently visualized and analyzed in PyMOL (v3.1) to assess structural features and potential interaction interfaces between the proteins [46].

## Results and discussion

Despite the well-established importance of G-patch proteins in modulating RNA helicase activity, the precise molecular functions of many members of this family remain incompletely understood. We therefore sought to determine whether the poorly characterized and essential fission yeast G-patch protein Cwf28 operates within a regulatory framework analogous to that described for its orthologs, *S. cerevisiae* Spp2 and human GPKOW [13,14,20,29,30].

**Fig 1:**
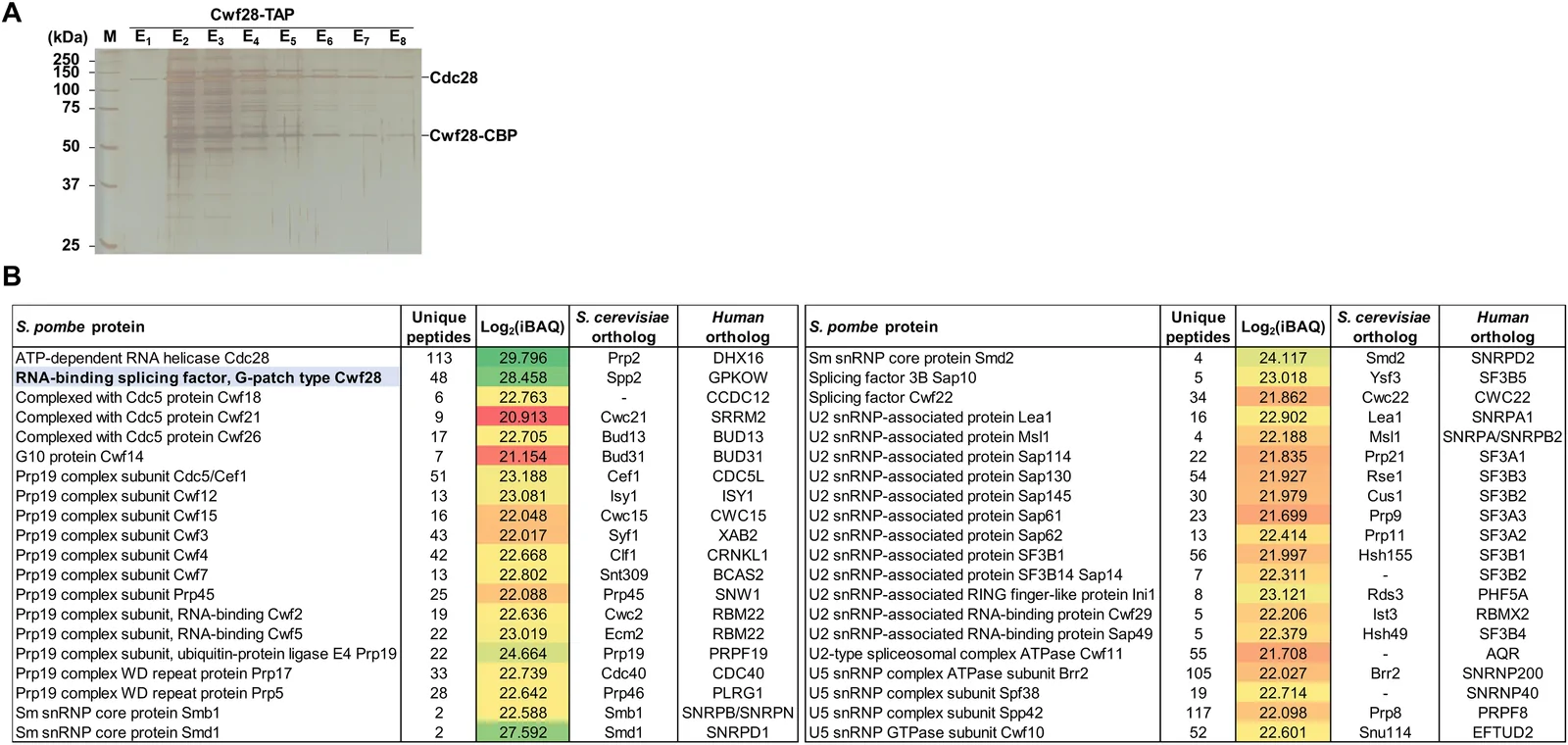
Identification of proteins co-purifying with G-patch protein Cwf28. (**A**) Proteins associated with Cwf28 were isolated by tandem affinity purification. The complexity of eluates (E_1_–E_8_) was analyzed by SDS-PAGE, and proteins were visualized by silver staining. Molecular weight marker (M) is indicated on the left. The positions corresponding to Cdc28 (~121 kDa) and the Cwf28-CBP fusion protein are indicated. (**B**) Selected proteins co-purifying with Cwf28 are shown. Proteins are ranked by their intensity-based absolute quantification (iBAQ) values from two independent biological replicates, providing an estimate of their relative abundance in the Cwf28 pulldown. Corresponding *S. cerevisiae* and human orthologs are indicated. For the complete list of identified proteins and quantitative information, see S1 Table.

To address this, we purified native Cwf28-containing complexes by tandem affinity purification and conducted comprehensive interactome analysis to identify associated factors (Fig 1A). Our results show that Cwf28 predominantly co-purifies with components of the NTC as well as proteins involved in the catalytic activation and progression of the spliceosome. Notably, the DEAH-box RNA helicase Cdc28, the ortholog of human DHX16 and *S. cerevisiae* Prp2, was identified as the most abundant and prominent interactor of Cwf28 (Fig 1B and S1 Table).

Consistent with previous characterization and annotation of spliceosomal components in *S. pombe* [15], only a limited subset of spliceosome-associated factors, including NTC-associated proteins and U2-type catalytic spliceosome-associated factors (Cwf16, Cwf19, Cwf25, and Mug161), were not detected among the proteins co-purifying with Cwf28. In addition, components of the casein kinase 2 (CK2) complex, including the catalytic subunit Cka1 and the regulatory subunits Ckb1 and Ckb2 [47], were identified in the Cwf28 interactome (S1 Table). The presence of the CK2 complex agrees with earlier studies demonstrating its association with spliceosomal assemblies in *S. pombe*, where it is thought to regulate the activity of spliceosome-associated factors through phosphorylation [48–51].

**Fig 2:**
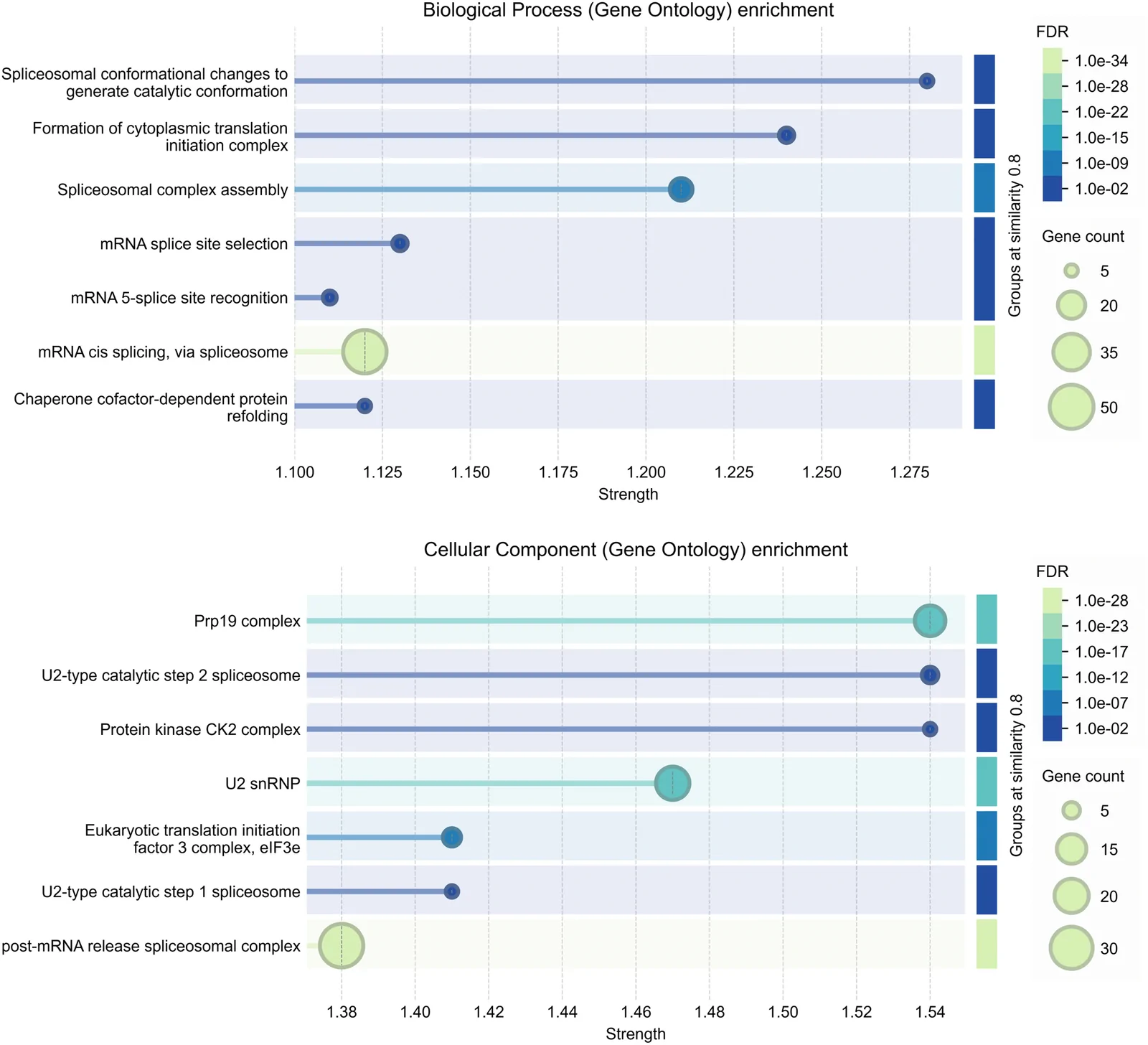
GO enrichment analysis of proteins co-purifying with Cwf28. The scatter plot includes the top 7 most enriched biological process and cellular component categories. The X-axis represents the strength (the enrichment effect), and the Y-axis shows the specific GO terms. The color of the dots represents the FDR values, with light green indicating lower FDR values (higher significance) and blue indicating higher FDR values (lower significance). Dot size corresponds to the number of proteins in the respective GO term.

Gene Ontology (GO) analysis revealed significant enrichment of proteins associated with biological processes related to pre-mRNA splicing, including spliceosomal conformational rearrangements leading to catalytic activation, spliceosomal complex assembly, mRNA splice site selection, 5′ splice site recognition, and mRNA splicing, among others. In terms of cellular components, the Cwf28 interactome was enriched for proteins associated with the NTC, U2-type catalytic step 1 and step 2 spliceosomes, the protein kinase CK2 complex, and others (Fig 2). Collectively, these results strongly indicate that the G-patch protein Cwf28 is an integral component of catalytically active spliceosomal assemblies and likely functions in coordinating spliceosome activation and remodeling, potentially through regulation of RNA helicase Cdc28.

Interestingly, multiple phosphorylation sites were identified among proteins co-purifying with Cwf28, including S95, S96, and S138 of Cdc28; S535 of Cip2; S385 of Cwf2; T62, S721, and S853 of Cwf22; S11, S13, S31, S101, S102, S152, S277 of Cwf28; S160 of Cwf29; S51 of Prp10; S39 of Ctnnbl1; and S228 and S236 of Prp45 (S1 Table). The phosphorylation events detected within Cwf28-associated complexes indicate that multiple spliceosomal factors associated with Cwf28 are phosphorylated. Notably, the CK2 complex was identified among the Cwf28-associated proteins, and several of the identified phosphosites correspond to residues predicted to be compatible with CK2 substrate preferences, including S51 of Prp10, S39 of Ctnnbl1, S385 of Cwf2, S721 of Cwf22, S96 and S138 of Cdc28, and S11, S13, and S102 of Cwf28. These observations suggest a potential association between the CK2 complex and phosphorylation of Cwf28-associated spliceosomal components. However, the relative abundance and phosphorylation occupancy of these sites were not determined, and whether these modifications are directly mediated by the CK2 complex or have functional consequences for spliceosome function remains to be established. Future studies will be needed to define the contribution of individual phosphorylation events to spliceosome dynamics, RNA helicase activity, and pre-mRNA splicing efficiency and fidelity.

**Fig 3:**
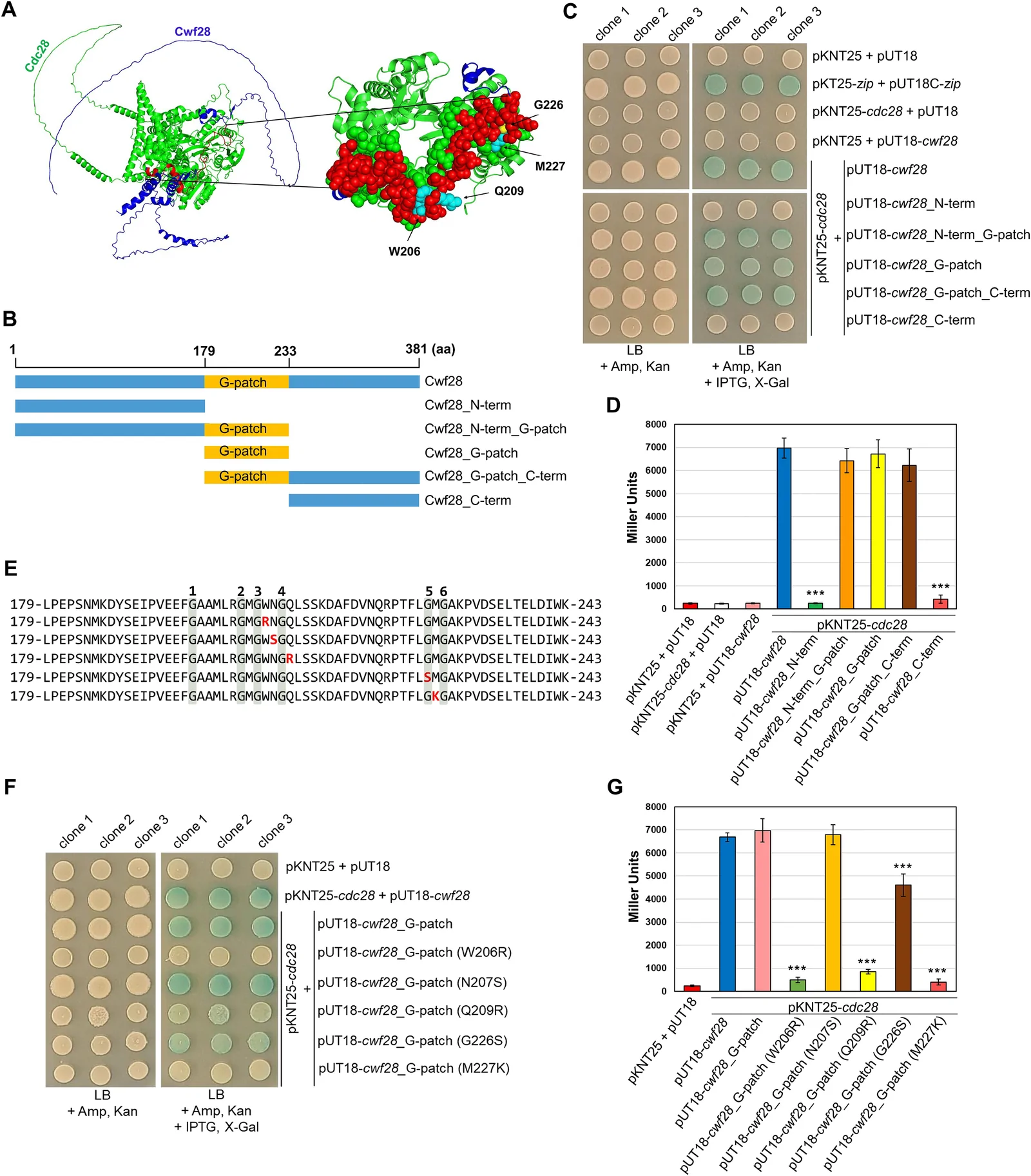
Analysis of interactions between the G-patch protein Cwf28 and the RNA helicase Cdc28. (**A**) AlphaFold-Multimer predicted the three-dimensional structure of the Cwf28–Cdc28 complex. Cwf28 is shown in blue, with the G-patch domain highlighted in red, while Cdc28 is depicted in green. The predicted Cwf28–Cdc28 interaction interface is shown in an enlarged view, with substituted amino acids within the G-patch domain labelled and colour-coded according to their effects on the interaction observed in the BACTH assay. The G-patch domain is shown in red, residues whose substitution abolished the interaction are indicated in cyan, and the residue whose substitution reduced the interaction is indicated in yellow. This model represents a predicted interaction surface that is supported by the BACTH results. (**B**) Schematic representation of specific Cwf28 constructs. (**C**) Alleles encoding Cdc28, Cwf28, and specific Cwf28 constructs were cloned into the pUT18 and pKNT25 plasmids of the BACTH assay. Empty pUT18 and pKNT25 vectors were used as negative controls, while pUT18C-zip and pKT25-zip plasmids served as positive controls. (**D**) Interaction strengths between the studied proteins and constructs were assessed by β-galactosidase activity assay and quantified in Miller units. Data represent mean ± S.D. from six independent biological replicates. (**E**) Schematic representation of specific Cwf28 G-patch domain mutants. Introduced single amino acid substitutions in the G-patch domain are indicated in red. Conserved glycine residues are highlighted in grey. (**F**) Alleles encoding Cdc28, Cwf28, and Cwf28 constructs containing specific single amino acid substitutions within the G-patch domain were assembled into the pUT18 and pKNT25 plasmids of the BACTH assay. Empty pUT18 and pKNT25 vectors were used as negative controls. (**G**) Interaction strengths between the studied proteins and constructs were assessed by β-galactosidase activity assay and quantified in Miller units. Data represent mean ± S.D. from six independent biological replicates. *Note:* Statistical significance of interactions between Cdc28 and Cwf28 G-patch domain constructs was evaluated relative to the full-length Cwf28 construct using an unpaired *t*-test (***p < 0.001).

The identification of the RNA helicase Cdc28 as the most abundant interactor within the Cwf28 complex (Figs 1A and 1B, S1 Table) prompted us to test whether these two proteins physically interact. Previous studies in *S. cerevisiae* demonstrated that the G-patch protein Spp2 interacts with and regulates the spliceosomal helicase Prp2 [13,14,29,30,52]. This interaction is mediated by the G-patch domain of Spp2 and involves the C-terminal region of Prp2, including the OB-fold motif [30]. To gain structural insight into the interaction between Cwf28 and Cdc28 in *S. pombe*, we employed AlphaFold-Multimer to predict the architecture of their complex. The resulting model positioned the G-patch domain of Cwf28 at the interface with Cdc28, where it adopts an extended conformation that engages a defined surface within the C-terminal region of the helicase, forming a putative interaction interface enriched in both hydrophobic and polar contacts (Fig 3A). This predicted arrangement resembles the interaction mode described for the *S. cerevisiae* Spp2–Prp2 complex, in which the G-patch domain contacts the C-terminal region of the helicase [30].

To experimentally validate this predicted interaction, we employed a bacterial adenylate cyclase two-hybrid (BACTH) assay. Consistent with the structural model, the BACTH results confirmed an interaction between Cwf28 and Cdc28 and further demonstrated that the G-patch domain is necessary and sufficient for this association. Notably, Cdc28 interaction was retained with all Cwf28-derived constructs containing the G-patch domain, including the N-terminal region encompassing the G-patch, the G-patch fused to the C-terminal region, and the G-patch domain alone. In contrast, no interaction was detected with Cwf28 constructs comprising only the N-terminal or C-terminal regions lacking the G-patch domain. These findings support the conclusion that the G-patch domain of Cwf28 is necessary for mediating interaction with Cdc28 (Figs 3B, 3C, and 3D).

To further elucidate the molecular basis of the interaction between the G-patch domain of Cwf28 and Cdc28, we generated a series of point mutations targeting specific residues within the G-patch sequence. Specifically, single amino acid substitutions were introduced at positions predicted to contribute to protein–protein interactions: W206R and N207S (immediately following the third conserved glycine), Q209R (following the fourth glycine), and G226S (fifth glycine) and M227K (adjacent to the fifth glycine residue) (Fig 3E). Assessing the impact of these substitutions on the interaction with Cdc28, we found that the G226S mutation resulted in a marked reduction in interaction strength, whereas the W206R, Q209R, and M227K substitutions abolished the interaction. In contrast, the N207S substitution did not produce a significant change in interaction compared to the wild-type G-patch domain (Figs 3F and 3G). The disruptive effects of W206R, Q209R, and M227K substitutions can be explained by alterations in physicochemical properties. Replacement of hydrophobic or aromatic residues (e.g., tryptophan at position 206 and methionine at position 227) with positively charged residues (arginine or lysine) likely disrupts hydrophobic contacts essential for stable binding. Additionally, substitution of glutamine with arginine (Q209R) introduces a charged side chain that may perturb local hydrogen-bonding networks or introduce electrostatic repulsion at the interaction interface. The moderate effect observed for G226S may reflect the importance of glycine in maintaining backbone flexibility. Its substitution with serine could restrict conformational adaptability required for optimal binding. In contrast, the N207S substitution represents a conservative change between polar residues, likely preserving hydrogen-bonding capacity and the overall structural integrity of the interaction interface. Although these results support the importance of specific G-patch residues in mediating the Cwf28–Cdc28 interaction, we cannot completely exclude the possibility that differences in fusion protein abundance may contribute to the observed interaction phenotypes, as a general limitation of the BACTH assay.

Collectively, the BACTH analysis demonstrates that the interaction between Cwf28 and Cdc28 is mediated specifically by the G-patch domain of Cwf28, supporting a model in which Cwf28 directly engages the RNA helicase Cdc28 through a defined G-patch-dependent interface. Consistent with both the mutational analysis and structural predictions, the G-patch domain of Cwf28 provides the essential interaction surface required for formation of the Cwf28–Cdc28 complex. This mode of interaction is consistent with previous studies in *S. cerevisiae* demonstrating that the G-patch protein Spp2 interacts with and modulates the enzymatic properties of the spliceosomal helicase Prp2 [13,14,29,30,52], similar to the regulation of the helicase Prp43 by the G-patch proteins Ntr1 and Pfa1 [28,53–55].

Given the established role of G-patch proteins as cofactors of DEAH-box helicases, our findings suggest that Cwf28 may contribute to the regulation or modulation of Cdc28 function during spliceosome progression in *S. pombe*. However, the direct impact of Cwf28 on Cdc28 activity remains to be determined. Thus, while our study positions Cwf28 in close association with catalytically active spliceosomal complexes and supports a functional relationship between Cwf28 and Cdc28, further biochemical and functional studies will be required to establish whether Cwf28 acts as a regulatory cofactor of the Cdc28 helicase. In particular, it will be important to determine whether the Cwf28–Cdc28 interaction is required for efficient and accurate pre-mRNA splicing, and how its disruption affects spliceosome dynamics, RNA processing fidelity, and overall cellular homeostasis. Future studies combining in vivo functional assays with high-resolution structural and biochemical approaches will be essential to further define the mechanistic contribution of Cwf28 to spliceosome function.

## Supporting information

S1 Table Primers, proteins identified in Cwf28-TAP purification, and their associated phosphorylation sites. (XLSX)

S1 Fig Original, uncropped images corresponding to Figs. 1A, 3C, and 3F. (TIF)

## References

[1] Wahl MC, Will CL, Lührmann R. The spliceosome: design principles of a dynamic RNP machine. Cell. 2009;136(4):701–18. doi: 10.1016/j.cell.2009.02.009 19239890

[2] Wilkinson ME, Charenton C, Nagai K. RNA Splicing by the Spliceosome. Annu Rev Biochem. 2020;89:359–88. doi: 10.1146/annurev-biochem-091719-064225 31794245

[3] Stepankiw N, Raghavan M, Fogarty EA, Grimson A, Pleiss JA. Widespread alternative and aberrant splicing revealed by lariat sequencing. Nucleic Acids Res. 2015;43(17):8488–501. doi: 10.1093/nar/gkv763 26261211 PMC4787815

[4] Bitton DA, Atkinson SR, Rallis C, Smith GC, Ellis DA, Chen YYC, et al. Widespread exon skipping triggers degradation by nuclear RNA surveillance in fission yeast. Genome Res. 2015;25(6):884–96. doi: 10.1101/gr.185371.114 25883323 PMC4448684

[5] Borao S, Ayté J, Hümmer S. Evolution of the Early Spliceosomal Complex-From Constitutive to Regulated Splicing. Int J Mol Sci. 2021;22(22):12444. doi: 10.3390/ijms222212444 34830325 PMC8624252

[6] Egecioglu DE, Chanfreau G. Proofreading and spellchecking: a two-tier strategy for pre-mRNA splicing quality control. RNA. 2011;17(3):383–9. doi: 10.1261/rna.2454711 21205840 PMC3039138

[7] Selicky T, Jurcik M, Mikolaskova B, Pitelova A, Mayerova N, Kretova M, et al. Defining the Functional Interactome of Spliceosome-Associated G-Patch Protein Gpl1 in the Fission Yeast Schizosaccharomyces pombe. Int J Mol Sci. 2022;23(21):12800. doi: 10.3390/ijms232112800 36361590 PMC9658070

[8] Soni K, Horvath A, Dybkov O, Schwan M, Trakansuebkul S, Flemming D, et al. Structures of aberrant spliceosome intermediates on their way to disassembly. Nat Struct Mol Biol. 2025;32(5):914–25. doi: 10.1038/s41594-024-01480-7 39833470 PMC12086092

[9] Li Y, Fischer P, Wang M, Zhou Q, Song A, Yuan R, et al. Structural insights into spliceosome fidelity: DHX35-GPATCH1- mediated rejection of aberrant splicing substrates. Cell Res. 2025;35(4):296–308. doi: 10.1038/s41422-025-01084-w 40016598 PMC11958768

[10] Sloan KE, Bohnsack MT. Unravelling the Mechanisms of RNA Helicase Regulation. Trends Biochem Sci. 2018;43(4):237–50. doi: 10.1016/j.tibs.2018.02.001 29486979

[11] Gencheva M, Kato M, Newo ANS, Lin R-J. Contribution of DEAH-box protein DHX16 in human pre-mRNA splicing. Biochem J. 2010;429(1):25–32. doi: 10.1042/BJ20100266 20423332 PMC3137565

[12] Zhan X, Yan C, Zhang X, Lei J, Shi Y. Structures of the human pre-catalytic spliceosome and its precursor spliceosome. Cell Res. 2018;28(12):1129–40. doi: 10.1038/s41422-018-0094-7 30315277 PMC6274647

[13] Bai R, Wan R, Yan C, Jia Q, Lei J, Shi Y. Mechanism of spliceosome remodeling by the ATPase/helicase Prp2 and its coactivator Spp2. Science. 2021;371(6525):eabe8863. doi: 10.1126/science.abe8863 33243853

[14] Warkocki Z, Schneider C, Mozaffari-Jovin S, Schmitzová J, Höbartner C, Fabrizio P, et al. The G-patch protein Spp2 couples the spliceosome-stimulated ATPase activity of the DEAH-box protein Prp2 to catalytic activation of the spliceosome. Genes Dev. 2015;29(1):94–107. doi: 10.1101/gad.253070.114 25561498 PMC4285774

[15] Wood V, Harris MA, McDowall MD, Rutherford K, Vaughan BW, Staines DM, et al. PomBase: a comprehensive online resource for fission yeast. Nucleic Acids Res. 2012;40(Database issue):D695-9. doi: 10.1093/nar/gkr853 22039153 PMC3245111

[16] Studer MK, Ivanović L, Weber ME, Marti S, Jonas S. Structural basis for DEAH-helicase activation by G-patch proteins. Proc Natl Acad Sci U S A. 2020;117(13):7159–70. doi: 10.1073/pnas.1913880117 32179686 PMC7132122

[17] Bohnsack KE, Ficner R, Bohnsack MT, Jonas S. Regulation of DEAH-box RNA helicases by G-patch proteins. Biol Chem. 2021;402(5):561–79. doi: 10.1515/hsz-2020-0338 33857358

[18] Karika LO, Cipakova I, Hronska L, Cipak L. G-patch proteins: important regulators of pre-mRNA splicing and ribosome biogenesis. Front Cell Dev Biol. 2026;14:1750689. 42052570 10.3389/fcell.2026.1750689 PMC13111387

[19] Chen Z. DEAH-Box RNA Helicases in the Spliceosome: Advances in Structure and Function. FASEB J. 2026;40(4):e71588. doi: 10.1096/fj.202503744R 41689426

[20] Zang S, Lin TY, Chen X, Gencheva M, Newo AN, Yang L, et al. GPKOW is essential for pre-mRNA splicing in vitro and suppresses splicing defect caused by dominant-negative DHX16 mutation in vivo. Biosci Rep. 2014; 34: e00163. 10.1042/BSR20140142 PMC4266926 25296192

[21] Lin M-L, Fukukawa C, Park J-H, Naito K, Kijima K, Shimo A, et al. Involvement of G-patch domain containing 2 overexpression in breast carcinogenesis. Cancer Sci. 2009;100(8):1443–50. doi: 10.1111/j.1349-7006.2009.01185.x 19432882 PMC11158124

[22] Ren T, Wei G, Yi J, Zhang Y, Zhao H, Wu N, et al. GPATCH3, a splicing regulator that facilitates tumor immune evasion via the modulation of ATPase activity of DHX15. Front Immunol. 2025;16:1612461. doi: 10.3389/fimmu.2025.1612461 40861452 PMC12375585

[23] Benbarche S, Pineda JMB, Galvis LB, Biswas J, Liu B, Wang E, et al. GPATCH8 modulates mutant SF3B1 mis-splicing and pathogenicity in hematologic malignancies. Mol Cell. 2024;84(10):1886-1903.e10. doi: 10.1016/j.molcel.2024.04.006 38688280 PMC11102302

[24] Vorländer MK, Rothe P, Kleifeld J, Cormack ED, Veleti L, Riabov-Bassat D, et al. Mechanism for the initiation of spliceosome disassembly. Nature. 2024;632(8024):443–50. doi: 10.1038/s41586-024-07741-1 38925148 PMC7616679

[25] Niu Z, Jin W, Zhang L, Li X. Tumor suppressor RBM5 directly interacts with the DExD/H-box protein DHX15 and stimulates its helicase activity. FEBS Lett. 2012;586(7):977–83. doi: 10.1016/j.febslet.2012.02.052 22569250

[26] Feng Q, Krick K, Chu J, Burge CB. Splicing quality control mediated by DHX15 and its G-patch activator SUGP1. Cell Rep. 2023;42(10):113223. doi: 10.1016/j.celrep.2023.113223 37805921 PMC10842378

[27] Heininger AU, Hackert P, Andreou AZ, Boon K-L, Memet I, Prior M, et al. Protein cofactor competition regulates the action of a multifunctional RNA helicase in different pathways. RNA Biol. 2016;13(3):320–30. doi: 10.1080/15476286.2016.1142038 26821976 PMC4829300

[28] Fourmann J-B, Tauchert MJ, Ficner R, Fabrizio P, Lührmann R. Regulation of Prp43-mediated disassembly of spliceosomes by its cofactors Ntr1 and Ntr2. Nucleic Acids Res. 2017;45(7):4068–80. doi: 10.1093/nar/gkw1225 27923990 PMC5397206

[29] Roy J, Kim K, Maddock JR, Anthony JG, Woolford JL Jr. The final stages of spliceosome maturation require Spp2p that can interact with the DEAH box protein Prp2p and promote step 1 of splicing. RNA. 1995;1(4):375–90. 7493316 PMC1482403

[30] Silverman EJ, Maeda A, Wei J, Smith P, Beggs JD, Lin R-J. Interaction between a G-patch protein and a spliceosomal DEXD/H-box ATPase that is critical for splicing. Mol Cell Biol. 2004;24(23):10101–10. doi: 10.1128/MCB.24.23.10101-10110.2004 15542821 PMC529041

[31] Cipakova I, Jurcik M, Rubintova V, Borbova M, Mikolaskova B, Jurcik J, et al. Identification of proteins associated with splicing factors Ntr1, Ntr2, Brr2 and Gpl1 in the fission yeast Schizosaccharomyces pombe. Cell Cycle. 2019;18(14):1532–6. doi: 10.1080/15384101.2019.1632126 31219728 PMC6619935

[32] Cipakova I, Karika LO, Hronska L, Selicky T, Iaparov B, Karhanek M, et al. GPATCH11 ortholog Sap34 regulates pre-mRNA splicing by interacting with early spliceosomal complexes in Schizosaccharomyces pombe. Sci Rep. 2026;16(1):26107. doi: 10.1038/s41598-026-57150-9 42260140 PMC13490427

[33] Carnahan RH, Feoktistova A, Ren L, Niessen S, Yates JR 3rd, Gould KL. Dim1p is required for efficient splicing and export of mRNA encoding lid1p, a component of the fission yeast anaphase-promoting complex. Eukaryot Cell. 2005;4(3):577–87. doi: 10.1128/EC.4.3.577-587.2005 15755920 PMC1087801

[34] Kallgren SP, Andrews S, Tadeo X, Hou H, Moresco JJ, Tu PG, et al. The proper splicing of RNAi factors is critical for pericentric heterochromatin assembly in fission yeast. PLoS Genet. 2014;10(5):e1004334. doi: 10.1371/journal.pgen.1004334 24874881 PMC4038458

[35] Cipak L, Spirek M, Novatchkova M, Chen Z, Rumpf C, Lugmayr W, et al. An improved strategy for tandem affinity purification-tagging of Schizosaccharomyces pombe genes. Proteomics. 2009;9(20):4825–8. doi: 10.1002/pmic.200800948 19750511 PMC2956173

[36] Cipak L, Selicky T, Jurcik J, Cipakova I, Osadska M, Lukacova V, et al. Tandem affinity purification protocol for isolation of protein complexes from Schizosaccharomyces pombe. STAR Protoc. 2022;3(1):101137. doi: 10.1016/j.xpro.2022.101137 35128479 PMC8808283

[37] Rabilloud T, Carpentier G, Tarroux P. Improvement and simplification of low-background silver staining of proteins by using sodium dithionite. Electrophoresis. 1988;9(6):288–91. doi: 10.1002/elps.1150090608 2466660

[38] Cox J, Mann M. MaxQuant enables high peptide identification rates, individualized p.p.b.-range mass accuracies and proteome-wide protein quantification. Nat Biotechnol. 2008;26:1367–72. 19029910 10.1038/nbt.1511

[39] Blom N, Gammeltoft S, Brunak S. Sequence and structure-based prediction of eukaryotic protein phosphorylation sites. J Mol Biol. 1999;294(5):1351–62. doi: 10.1006/jmbi.1999.3310 10600390

[40] Szklarczyk D, Nastou K, Koutrouli M, Kirsch R, Mehryary F, Hachilif R, et al. The STRING database in 2025: protein networks with directionality of regulation. Nucleic Acids Res. 2025;53(D1):D730–7. doi: 10.1093/nar/gkae1113 39558183 PMC11701646

[41] Wilson DS, Keefe AD. Random mutagenesis by PCR. Curr Protoc Mol Biol. 2001;Chapter 8:Unit8.3. doi: 10.1002/0471142727.mb0803s51 18265275

[42] Karimova G, Pidoux J, Ullmann A, Ladant D. A bacterial two-hybrid system based on a reconstituted signal transduction pathway. Proc Natl Acad Sci U S A. 1998;95(10):5752–6. doi: 10.1073/pnas.95.10.5752 9576956 PMC20451

[43] Karimova G, Ullmann A, Ladant D. A bacterial two-hybrid system that exploits a cAMP signaling cascade in Escherichia coli. Methods Enzymol. 2000;328:59–73. doi: 10.1016/s0076-6879(00)28390-0 11075338

[44] Jumper J, Evans R, Pritzel A, Green T, Figurnov M, Ronneberger O, et al. Highly accurate protein structure prediction with AlphaFold. Nature. 2021;596(7873):583–9. doi: 10.1038/s41586-021-03819-2 34265844 PMC8371605

[45] Mirdita M, Schütze K, Moriwaki Y, Heo L, Ovchinnikov S, Steinegger M. ColabFold: making protein folding accessible to all. Nat Methods. 2022;19(6):679–82. doi: 10.1038/s41592-022-01488-1 35637307 PMC9184281

[46] DeLano WL. The PyMOL Molecular Graphics System. Palo Alto, Ca, USA: DeLano Scientific. 2002.

[47] Cipak L, Gupta S, Rajovic I, Jin Q-W, Anrather D, Ammerer G, et al. Crosstalk between casein kinase II and Ste20-related kinase Nak1. Cell Cycle. 2013;12(6):884–8. doi: 10.4161/cc.24095 23462181 PMC3637346

[48] Lehnert S, Götz C, Kartarius S, Schäfer B, Montenarh M. Protein kinase CK2 interacts with the splicing factor hPrp3p. Oncogene. 2008;27(17):2390–400. doi: 10.1038/sj.onc.1210882 18026141

[49] Yan C, Hang J, Wan R, Huang M, Wong CCL, Shi Y. Structure of a yeast spliceosome at 3.6-angstrom resolution. Science. 2015;349(6253):1182–91. doi: 10.1126/science.aac7629 26292707

[50] Mikolaskova B, Jurcik M, Cipakova I, Selicky T, Jurcik J, Polakova SB, et al. Identification of Nrl1 Domains Responsible for Interactions with RNA-Processing Factors and Regulation of Nrl1 Function by Phosphorylation. Int J Mol Sci. 2021;22(13):7011. doi: 10.3390/ijms22137011 34209806 PMC8268110

[51] Jurcik J, Cipakova I, Karika LO, Bellova J, Kohutova L, Gregan J, et al. Exploring the Cka1 kinase interactome: unveiling novel potential regulatory roles of CKII kinase in S. pombe. Biochem Biophys Res Commun. 2025;778:152382. doi: 10.1016/j.bbrc.2025.152382 40700808

[52] Wlodaver AM, Staley JP. The DExD/H-box ATPase Prp2p destabilizes and proofreads the catalytic RNA core of the spliceosome. RNA. 2014;20(3):282–94. doi: 10.1261/rna.042598.113 24442613 PMC3923124

[53] Tanaka N, Aronova A, Schwer B. Ntr1 activates the Prp43 helicase to trigger release of lariat-intron from the spliceosome. Genes Dev. 2007;21(18):2312–25. doi: 10.1101/gad.1580507 17875666 PMC1973145

[54] Lebaron S, Papin C, Capeyrou R, Chen Y-L, Froment C, Monsarrat B, et al. The ATPase and helicase activities of Prp43p are stimulated by the G-patch protein Pfa1p during yeast ribosome biogenesis. EMBO J. 2009;28(24):3808–19. doi: 10.1038/emboj.2009.335 19927118 PMC2797057

[55] Walbott H, Mouffok S, Capeyrou R, Lebaron S, Humbert O, van Tilbeurgh H, et al. Prp43p contains a processive helicase structural architecture with a specific regulatory domain. EMBO J. 2010;29(13):2194–204. doi: 10.1038/emboj.2010.102 20512115 PMC2905241

